# Innovation through Artificial Intelligence in Triage Systems for Resource Optimization in Future Pandemics

**DOI:** 10.3390/biomimetics9070440

**Published:** 2024-07-18

**Authors:** Nicolás J. Garrido, Félix González-Martínez, Susana Losada, Adrián Plaza, Eneida del Olmo, Jorge Mateo

**Affiliations:** 1 Internal Medicine, Virgen de la Luz Hospital, 16002 Cuenca, Spain; 2 Expert Medical Analysis Group, Institute of Technology, University of Castilla-La Mancha, 16071 Cuenca, Spain; 3 Department of Emergency Medicine, Virgen de la Luz Hospital, 16002 Cuenca, Spain; 4 Expert Medical Analysis Group, Instituto de Investigación Sanitaria de Castilla-La Mancha (IDISCAM), 45071 Toledo, Spain

**Keywords:** emergency, predictive value of tests, hospital mortality, machine learning, pandemics

## Abstract

Artificial intelligence (AI) systems are already being used in various healthcare areas. Similarly, they can offer many advantages in hospital emergency services. The objective of this work is to demonstrate that through the novel use of AI, a trained system can be developed to detect patients at potential risk of infection in a new pandemic more quickly than standardized triage systems. This identification would occur in the emergency department, thus allowing for the early implementation of organizational preventive measures to block the chain of transmission. Materials and Methods: In this study, we propose the use of a machine learning system in emergency department triage during pandemics to detect patients at the highest risk of death and infection using the COVID-19 era as an example, where rapid decision making and comprehensive support have becoming increasingly crucial. All patients who consecutively presented to the emergency department were included, and more than 89 variables were automatically analyzed using the extreme gradient boosting (XGB) algorithm. Results: The XGB system demonstrated the highest balanced accuracy at 91.61%. Additionally, it obtained results more quickly than traditional triage systems. The variables that most influenced mortality prediction were procalcitonin level, age, and oxygen saturation, followed by lactate dehydrogenase (LDH) level, C-reactive protein, the presence of interstitial infiltrates on chest X-ray, and D-dimer. Our system also identified the importance of oxygen therapy in these patients. Conclusions: These results highlight that XGB is a useful and novel tool in triage systems for guiding the care pathway in future pandemics, thus following the example set by the well-known COVID-19 pandemic.

## 1. Introduction

Artificial intelligence (AI) is currently revolutionizing many fields, including medicine and especially emergency services, by enhancing human diagnostic capabilities, thus optimizing resource allocation and ultimately improving patient outcomes. In recent years, AI technologies have shown significant advancements, particularly in speeding up and improving decision-making processes, which can be crucial in emergency settings where precise decisions can significantly impact patient survival and subsequent recovery [1,2].

In the specific case of COVID-19, it is a respiratory disease caused by a coronavirus popularly known as SARS-CoV-2, which was declared a public health emergency of international concern by the World Health Organization (WHO) in January 2020 [3]. Since that moment, our efforts have been directed towards discovering the variables associated with severe disease, thus identifying all comorbidities to select the best therapy [4,5,6,7], developing effective treatments [8,9,10,11,12,13], and creating classifications to quickly assess potentially severe patients. For example, the National Early Warning Score (NEWS) was created in emergency services as a good predictor of ICU admission within 7 days [14], as well as the Rapid Emergency Medicine Score (REMS) to predict death within 7 days [15]. At some point during the pandemic, these scales were used to prioritize the most critical patients for transfer to another center with more specific care [16].

After this experience, it has become evident that during a pandemic, the burden on hospitals worldwide increases while resources are limited. To reduce transmission, direct contact with many patients is limited as much as possible, and in this context, AI systems can help triage according to severity to determine, for example, which patient mandatorily needs a heart transplant [17].

Additionally, the COVID-19 era has been leveraged to attempt to compare the risk factors and clinical characteristics of this virus with others, such as influenza [18], or to establish a degree of need for healthcare based on laboratory data [19], although the prognostic value of many of these factors remains uncertain.

Delving deeper, these AI systems are becoming increasingly complex and have begun to be applied during the COVID-19 era to provide faster initial diagnostic impressions from various imaging tests commonly used in medicine. They have been utilized by different ophthalmology services to diagnose age-related macular degeneration, many refractive errors, glaucoma, and even diabetic retinopathy [20].

Another development was the study of applying these AI techniques to chest X-ray and computed tomography (CT) images in emergency services to obtain automated and accurate solutions for COVID-19 detection [21,22,23,24]. All this aims to enhance the physician’s capacity, thus making the possibilities for the introduction of this technology in medicine limitless [25].

This capability will be very important in the coming years, as the increase in life expectancy in Europe and most developed countries will lead to a progressive rise in hospital visits. This will result in increased frailty, multimorbidity, and consequently, the risk of serious illness and mortality, with emergency services being the first to be impacted. In line with this, countries like the United States will be required to develop new models for the early detection of clinical deterioration, as the average mortality rate of hospitalized patients is around 2%, and recent studies suggest that some deaths resulting from such deterioration could have been prevented [26,27], especially in a new pandemic situation.

For the early detection of critical patients, vital signs such as pulse, blood pressure, heart rate, respiratory rate, body temperature, oxygen saturation, and the Glasgow Coma Scale remain fundamental in healthcare. These indicators are routinely collected during emergency admission processes, and despite being used on the front line for initial patient assessments and having been known in clinical practice for over a century, their effectiveness in various clinical scenarios has not been thoroughly evaluated. Recent research has confirmed that changes in vital signs often precede fatal outcomes by several hours. Currently, the measurement of vital signs plays a crucial role in identifying patients at risk of deterioration in emergency services. However, this deterioration can sometimes go unnoticed or is not detected until it is too late to intervene [28,29].

In critical patient care, as provided in an emergency service, it is imperative to act as quickly as possible, especially in a situation of overload due to a pandemic. In this context, the rapid identification and early isolation of infected patients can help block the spread of the disease to hospital staff and the community at large, thus making it even more important to have technology that speeds up the process [30,31]. Additionally, as previously mentioned, early intervention by the physician significantly improves patients’ survival prospects. In the case of severe infectious diseases, early treatment can also prevent future complications. Here, artificial intelligence could also prove beneficial. Another important point is to act swiftly to identify and quarantine individuals who have been in close contact with previously infected patients, thereby breaking the chain of transmission and controlling the spread of the virus [26,27].

Given that resources are limited (hospital beds, medical equipment, and personnel), a rapid response allows for more efficient management of these resources, thus ensuring that their allocation aligns with patients’ needs. In a situation of overload due to a pandemic, having AI systems that assist in a preliminary assessment of the patient’s severity for initial resource allocation would greatly optimize medical care. Furthermore, it is crucial to communicate the epidemiological situation to the public, along with preventive measures and behavior guidelines, to reduce anxiety and foster community cooperation in containing the pandemic. All this rapid action starts in emergency services, thus ultimately aiming to limit mortality and control the spread of the microorganism. Therefore, it is the perfect scenario in which the use of artificial intelligence tools can significantly improve disease detection, response, and management capabilities, thereby contributing to saving lives and reducing the impact of future health crises [2,32].

Moreover, there is increasing research into the possible causes of revisits to emergency services using artificial intelligence methods [2,33]. Machine learning, a branch of artificial intelligence, encompasses tools that examine datasets to detect patterns, thus continuously improving their capabilities as more data become available. Ultimately, these technologies are faster and more effective than traditional methods. In this way, several learning algorithms have been developed to address a wide range of challenges. Consequently, learning algorithms, particularly deep learning, are now used to identify individuals at risk. As highlighted by Agam Bansal, these advanced learning techniques have already been integrated into the fight against COVID-19, and they will undoubtedly regain significant importance in combating a new health crisis [34,35,36,37,38]. There are studies that could be attempted to apply for solving the COVID-19 equation that can be used to approximate its solutions using numerical methods [39,40,41,42].

The objective of this work is to demonstrate the utility of machine learning (ML) algorithms for patients attending hospital emergency services in immediately determining their vital prognosis. As a result, it has been validated as a reliable approach for identifying patterns in other medical conditions, such as systemic lupus erythematosus, coagulopathy induced by traumatic brain injuries, epilepsy, diabetes, Alzheimer’s disease, HIV, and various types of cancer. Thus, we used machine learning techniques to determine the most influential variables in predicting mortality in emergency services during the COVID-19 era, a period in which the population lacked herd immunity, and no prior vaccination existed. The analytical power, speed of obtaining results, and high accuracy indicate that this computational tool should be used in any emergency service to anticipate outcomes in future pandemics. The proposed system allows for the analysis of large epidemiological datasets to identify patterns associated with the disease and risk factors.

## 2. Materials and Methods

### Materials

This study was conducted in the emergency department of the Virgen de la Luz Hospital, as well as the reference hospital in the metropolitan area of Cuenca in the Castilla-La Mancha region. All consecutive patients who attended the emergency department between 2 March and 30 April 2020 were included. The cases were selected from patients over 18 years of age who presented with any symptoms associated with acute COVID-19 infection and tested positive for the SARS-CoV-2 polymerase chain reaction (PCR) test from a nasopharyngeal swab in the emergency department.

Patients who presented to the emergency department outside the study period, those under 18 years of age, individuals presenting with nonrespiratory symptoms, and patients for whom PCR testing could not be performed due to reasons such as patient refusal, anatomical abnormalities affecting nasopharyngeal sampling, leaving against medical advice, voluntary discharge, or discharge before testing were excluded.

Variable Selection: This single-center, observational, cross-sectional study involved obtaining data by reviewing the patients’ emergency medical records. An individual external to the research team tabulated and anonymized the data for all these patients.

A total of 89 variables were collected: registration number; three demographic variables (gender, nationality, and age); personal history (hypertension, diabetes mellitus, COPD, severe asthma, chronic kidney disease, obesity, pregnancy, dyslipidemia, liver disease, thromboembolic disease, active cancer, smoking, pharmacological immunosuppression, institutionalized, vascular disease, duration of illness); emergency department symptoms (cough, fever, dyspnea, chest pain, myalgia, headache, odynophagia, anosmia, ageusia, diarrhea, asthenia, Glasgow Coma Scale, asthenia, odynophagia, quick Sequential Organ Failure Assessment (qSOFA) score); hemodynamic variables (heart rate, baseline oxygen saturation, systolic and diastolic blood pressure, respiratory rate, temperature, fraction of inspired oxygen (FiO2)); date and duration of hospitalization; hospitalization outcome (discharge or death); 8 variables related to hospital treatment (oxygen therapy, antibiotics, hydroxychloroquine, low molecular weight heparin (LMWH); corticosteroids—methylprednisolone or dexamethasone; immunomodulators—anakinra, cyclosporine, tocilizumab, baricitinib; antivirals—lopinavir/ritonavir, emtricitabine/tenofovir disoproxil, darunavir/cobicistat, bronchodilators); 5 variables related to treatment after discharge (LMWH, methylprednisolone and dosage, dexamethasone and dosage); laboratory findings (hemoglobin, leukocytes, platelets, lymphocytes, D-dimer, prothrombin time (PT), creatinine, alanine transaminase, troponin, albumin, total proteins, ferritin, lactate dehydrogenase (LDH), C-reactive protein (CRP), procalcitonin); gasometric variables (pH, partial pressure of carbon dioxide (PCO2), partial pressure of oxygen (PO2), PO2/FiO2 ratio); 2 findings on emergency department radiography (unilateral infiltrate, bilateral infiltrate); and whether a chest computed tomography (CT) scan was performed.

Ethical Aspects: The study protocol was approved by the Clinical Research Ethics Committee of the Virgen de la Luz Hospital. We adhered to all principles of the Declaration of Helsinki and Law 15/99 on Data Protection, thus strictly maintaining patient anonymization. The physicians who collected the data were entirely separate from those who conducted the subsequent analysis.

## 3. Model Development

XGB is a predictive algorithm based on supervised learning. Technically, XGB employs boosting, iteratively correcting errors to capture complex interactions, offers robust L1 and L2 regularization to prevent overfitting, efficiently handles missing data, and is highly efficient in terms of computation. XGB adapts well to imbalanced data thanks to its ability to assign weights to observations and provides a clear mechanism for evaluating feature importance. The choice of XGB was based on its inherent technical advantages and its demonstrated empirical performance, thus offering an optimal balance between precision, efficiency, and the ability to handle complex data [43,44,45]. Owing to these characteristics, XGB was chosen to develop a classification system for COVID-19 patients in the emergency department. When presented with a dataset $S=\{x_{j},y_{j}\}$, the proposes model was devised as follows:

(1) $$\hat{y_{j}}=\phi \left(x_{j}\right)=\sum_{p=1}^{P}t_{p}\left(x_{j}\right).$$

In this context, $y_{j}$ represents the input consisting of *m* temporal variables, *P* denotes the total number of trees, $\hat{y_{j}}$ represents the predicted output, $x_{j}$ signifies the output, and $t_{p}$ stands for a tree with leaf weight $w_{p}$ and structure $u_{p}$, where *j* ranges from 1 to *n*.

The regularization target function for the suggested approach is detailed in Equation (2). Unlike standard Ensemble methods, this method employs a second-order Taylor expansion to approximate the XGB target function, thereby enhancing prediction accuracy [43,44,45].

(2) $$R\left(\phi \right)=\sum_{j}r\left(\hat{y_{j}},y_{j}\right)+\sum_{p}\Psi \left(t_{p}\right),$$

(3) $$\Psi \left(t_{p}\right)=\lambda f_{p}+\frac{1}{2}\gamma {∥w_{p}∥}^{2}.$$

In order to regulate the complexity (Ψ()) of the method and prevent overfitting, a regularization term, indicated by the weights, acts as a monitoring mechanism. As shown in Equation (3), $f_{p}$ represents tree pruning, which controls overfitting by indicating the number of leaves in the tree. The learning rate is denoted by λ, while *w* signifies the vector of scores assigned to the leaves. The function R() quantifies the disparity between the target output $y_{j}$ and the predicted output $\hat{y_{j}}$, thereby penalizing the method’s complexity. The parameter γ is used to adjust the weight of the system’s complexity [43,44,45]. This study aimed to enhance performance by minimizing Equation (2).

The tree set model incorporates the functionalities described in Equation (2). Therefore, optimization methods in Euclidean space cannot be used to optimize Equation (2). Instead, the model must be trained incrementally. In this study, $\hat{y_{j}}$ represents the estimate of the *j*th sample in the *s*th iteration. The goal is to find an additive function $C_{s}$ (representing the *s*th tree in the ensemble) that minimizes $R^{\left(s\right)}$:

(4) $$R^{\left(s\right)}=\sum_{j=1}r\left({\hat{y_{j}}}^{\left(s-1\right)},y_{j}+C_{s}\left(x_{j}\right)\right)+\Psi \left(C_{s}\right).$$

This is accomplished by sequentially adding trees to the ensemble, with each new tree being trained to correct the errors (residuals) of the previous ensemble. This additive method enables the model to progressively learn complex data patterns by concentrating on the residual errors from earlier predictions. The proposed model employs a second-order approximation to improve the target function, as suggested in [43,44,45].

(5) $$R^{\left(s\right)}\approx \sum_{j=1}\left[r\left({\hat{y_{j}}}^{\left(s-1\right)},y_{j}\right)+h_{j}C_{s}\left(x_{j}\right)+\frac{1}{2}b_{j}C_{s}^{2}\left(x_{j}\right)\right]+\Psi \left(C_{s}\right),$$

where $h_{j}$ represents the first-order gradient statistic with respect to ${\hat{y_{j}}}^{\left(s-1\right)}$ in the loss function R(), while $b_{j}$ denotes the second-order derivative with respect to ${\hat{y_{j}}}^{\left(s-1\right)}$ in the same context.

Since $r({\hat{y_{j}}}^{\left(s-1\right)},y_{j})$ remains constant, it can be removed to streamline Equation (5). If we define $K_{j}$ as the sample set of leaf *v*, where *v* ranges from 1 to fp, and expand the function Ψ(), Equation (5) can be represented as follows:

(6) $${\tilde{R}}^{\left(s\right)}\approx \sum_{j=1}\left[\sum_{j\in K_{v}}\left(h_{k}\right)w\_r_{v}+\frac{1}{2}\sum_{j\in K_{v}}\left(b_{j}+\gamma \right)w\_r_{v}^{2}\right]+\lambda F,$$

where ${\tilde{R}}^{\left(s\right)}$ signifies the simplified form of $R^{\left(s\right)}$ achieved by removing constant terms. The best weight $w\_r_{v}$ assigned to leaf *v* within a given structure u(x) can be calculated in the following manner:

(7) $$w\_r_{v}=-\frac{\sum_{j\in K_{v}}\left(h_{k}\right)}{\sum_{j\in K_{v}}\left(b_{j}+\gamma \right)}.$$

Ultimately, for the proposed approach, the optimal value can be attained through the following:

(8) $${\tilde{R}}^{\left(s\right)}\left(u\right)=-\frac{1}{2}\sum_{v=1}^{f_{p}}\frac{{\left(\sum_{j\in K_{v}}\left(h_{k}\right)\right)}^{2}}{\sum_{j\in K_{v}}\left(b_{j}+\gamma \right)}+\lambda F.$$

Initially, we conducted thorough data preprocessing to optimize the performance of our models. During data cleaning, we managed missing values using appropriate imputation techniques such as mean, median, or mode based on the data nature, and we addressed outliers through quartile analysis and boxplots to prevent negative impacts on the model performance. Subsequently, we encoded categorical variables using one-hot encoding for nominal variables and ordinal encoding to maintain the natural order of categories. Following this, numerical features were scaled using Min-Max normalization to ensure equitable contribution to the model. For class balancing in imbalanced datasets, we employed techniques like SMOTE for oversampling and undersampling methods. We then partitioned the dataset into training and testing sets using a typical 70–30 split while implementing 5-fold crossvalidation to robustly evaluate model performance and mitigate overfitting. These meticulous preprocessing steps were implemented to enhance the data quality and ensure that our machine learning models received relevant and well-prepared information, thus resulting in improved performance and enhanced model robustness, as evidenced by the metrics presented in our study.

**Figure 1:**
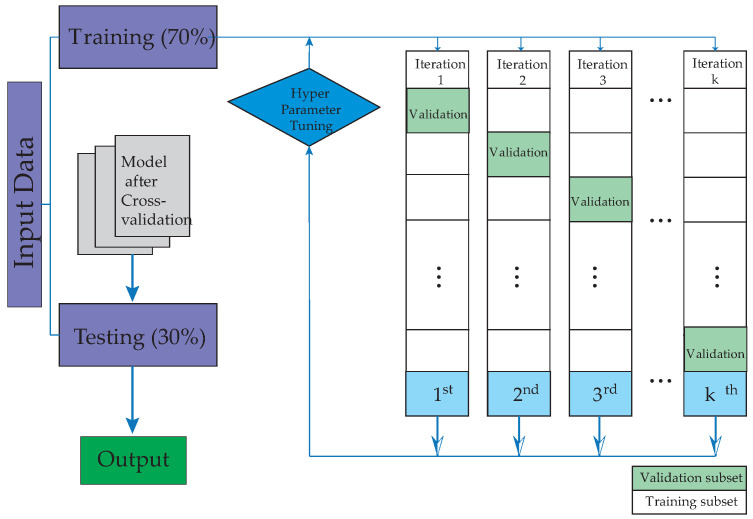
The illustration depicts the stages of learning and validation undertaken for machine learning algorithms.

This research subjected the proposed method to a comprehensive comparison with various machine learning techniques to classify the mortality of COVID-19 patients in the emergency department. The comparative analysis included Decision Trees (DTs) [46,47], Gaussian Naive Bayes (GNB) [48,49,50], K-Nearest Neighbors (KNNs) [51,52,53], Support Vector Machines (SVMs) [54,55,56], Neural Networks (NNs) [57,58], Random forest (RF) [59,60,61], the Convolutional Neural Network (CNN) [62,63], AdaBoost [64,65], and the novel method proposed in this study [66,67,68,69]. MATLAB software (MATLAB 2023a) was used for the assessment. To mitigate overfitting, a 5-fold crossvalidation strategy was employed. The dataset was divided into two parts, with 70% used for training and 30% for testing, thus ensuring that there was no overlap of patients between the two groups. The study workflow is schematically represented in Figure 1. It begins with patient selection and database creation, followed by the training phase, and it concludes with the validation of the implemented models.

Two performance metrics, namely accuracy and area under the curve (AUC), were used to evaluate and enhance the results. In addition, to mitigate the effects of randomness inherent in machine learning, the study conducted 100 random iterations. This approach aimed to reduce the influence of data noise, identify optimal parameters, and ensure statistically robust outcomes, as detailed in [70].

Machine learning techniques often rely on various hyperparameters to fine-tune the algorithm during training. These hyperparameters encompass settings such as the number of splits, types of learners, nearest neighbors, distance metrics, kernel types, box constraint levels, and methods for handling multiple classes, among others. The proper selection and adjustment of these hyperparameters are crucial, as they greatly influence the predictive accuracy and efficiency of the algorithm. Experimenting with different values of these hyperparameters is essential to achieve the best possible performance. In this study, Bayesian optimization was employed as a method to systematically refine and optimize the hyperparameters for each machine learning technique utilized.

Bayesian optimization seeks to find the best hyperparameter settings that maximize algorithm performance, thus building on past attempts while assuming a relationship between hyperparameters and performance metrics. Performance metrics such as the area under the receiver operating characteristic curve (AUC) and balanced accuracy were crucial in guiding this optimization process. Given the inherent randomness in machine learning and simulations, the study conducted 100 repetitions to compute the mean and standard deviation values of the performance metrics. To address data variability and enhance the reliability of the AUC values and statistical significance, experiments were repeated randomly and uniformly. The specific configurations used in this study are detailed in Table 1.

### Performance Evaluation

To analyze the performance of the compared algorithms, we employed the following measures to assess the accuracy: degenerate Youden index (DYI), receiver operating characteristic (ROC) curve, specificity, recall (also known as sensitivity), precision, and AUC [71].

## 4. Results

During the study period (2 March to 30 April 2020), over 13,000 patients presented to the emergency department with symptoms compatible with COVID-19. Among them, 708 patients tested positive for the SARS-CoV-2 PCR test. Figure 1 illustrates that 183 patients were excluded due to being under 18 years old. Of the included patients, 225 (37%) were male. The most prevalent comorbidities observed during their emergency department visits were hypertension (52%), type 2 diabetes mellitus (25%), and dyslipidemia (25%). Chronic obstructive pulmonary disease (COPD) (8.9%), obesity (7.4%), and chronic kidney disease (CKD) (6.6%) were also recorded. It is worth mentioning that in our sample, 4.5% of the patients had some form of active cancer, and eight patients were immunocompromised (1.3%). Less frequent comorbidities included liver diseases (1.5%) and previous thromboembolic events (1.5%). Other types of comorbidities were also recorded, such as nine patients (1.4%) with some form of liver disease. An overview of the sample characteristics is provided in Table 2.

Regarding symptoms, fever was the most prevalent (72.5%), followed by cough (62.5%), dyspnea (54%), asthenia (22%), myalgia (16%), and headache (1.6%). In our series, 19 patients initially presented with anosmia (3.1%), 26 patients with ageusia (4.3%), and 65 patients consulted for diarrhea (10%), thus confirming the great clinical variability of this virus. Of all patients with a positive PCR for SARS-CoV-2, 495 (81.8%) were admitted to the hospital, of whom 132 (21.8%) ultimately passed away.

The proposed XGB model was compared with various algorithms, and the system was trained to determine which achieved better mortality prediction, as well as the most important variables in the prediction. Standard parameters widely used in the scientific community were employed, as shown in Table 3 and Table 4. As shown in Table 3, the XGB model proposed in this study demonstrated superior performance compared to the other analyzed methods, thus achieving precision values approaching 91%. Specifically, it outperformed the RF algorithm in prediction by 2.48% and AdaBoost by 3.09%. GNB and BLDA exhibited the lowest accuracy among the tested systems.

Other metrics, including the area under the curve (AUC), Matthews correlation coefficient (MCC), degenerate Youden index (DYI), and Kappa index, were also assessed. The MCC is a robust statistical measure that yields a high score only if predictions perform well across all four categories of the confusion matrix (true positives, false negatives, true negatives, and false positives) proportionally to the distribution of positive and negative instances in the dataset. Table 4 illustrates that the XGB method proposed in this study achieved an MCC value nearing 1, thus indicating higher accuracy in predicting mortality compared to other methods. Another metric considered was the Kappa index, where the XGB system again outperformed RF and AdaBoost (Table 3).

**Figure 2:**
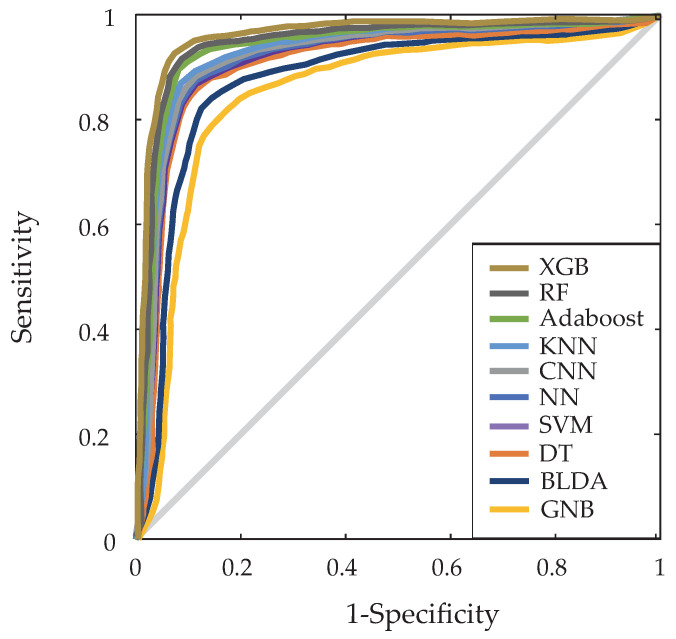
The figure illustrates the various ROC curves of the five machine learning algorithms that were analyzed.

We have also generated the receiver operating characteristic (ROC) curve to assess the performance of our proposed system against other ML methods. As can be observed in Figure 2, it displays the results obtained by various systems in predicting mortality variables. As depicted in Figure 2, the proposed XGB method exhibited a larger area under the curve (AUC) of 0.91, with the RF method (AUC of 0.89) being the closest in performance. Despite optimizing the RF model, the results consistently showed that XGB offered slightly superior performance in terms of key metrics such as precision, recall, F1 score, and AUC–ROC. This can be attributed to XGB’s inherent ability to handle complex feature interactions and its boosting approach, which iteratively corrects errors, something that RF, as a bagging-based ensemble method, cannot do in the same way. Additionally, XGB provides other advantages such as better handling of missing data, more precise control over overfitting through regularization parameters, and the ability to incorporate weights in observations, which is particularly useful in imbalanced datasets. In structured data, interpreting features is crucial to understanding how each variable contributes to model predictions. CNNs, designed to extract complex hierarchical features, may not be as transparent in feature interpretation as XGB models. XGB’s recursive partitioning of feature space makes it more efficient and faster to train on structured data compared to the iterative learning and feedback process characteristic of CNNs. Structured data often exhibit high dimensionality and sparsity, thus posing challenges for CNNs to effectively learn direct and efficient relationships between variables, which is why they did not achieve the precision values attained by XGB.

**Figure 3:**
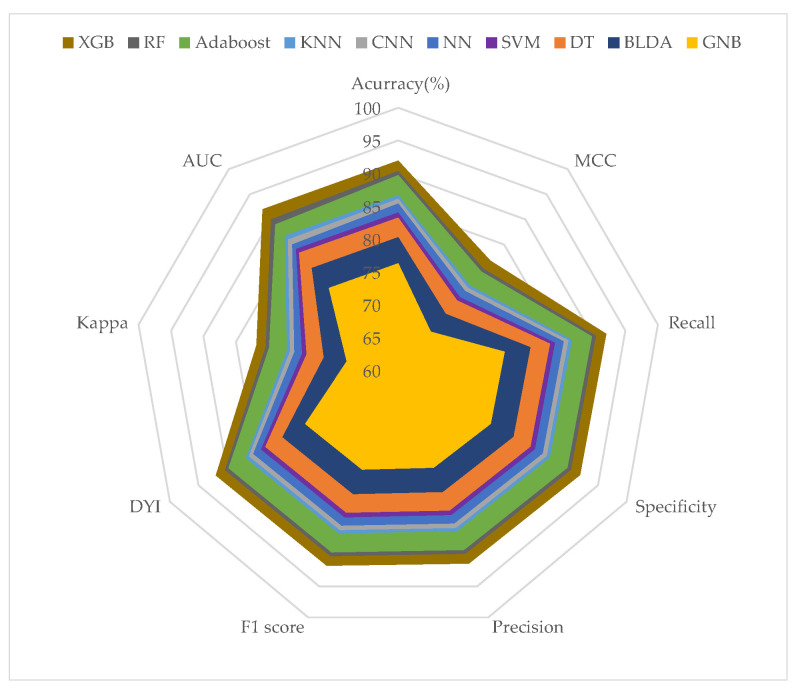
The figure shows the results of training phase in a radar graph.

**Figure 4:**
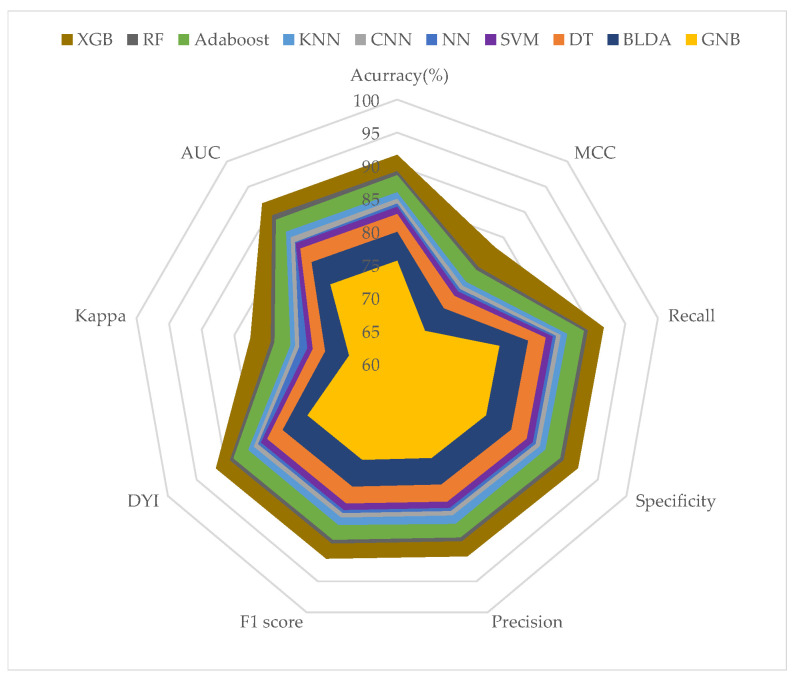
The figure displays the results of validation phase in a radar graph.

To visualize the results more effectively, we aggregated all measurements from each training dataset (Figure 3) and test dataset (Figure 4) and represented them using radar charts. Perfect performance across all measurements would be depicted as a circle covering the entire grid. To avoid overtraining, the test dataset results should not significantly differ from the training dataset results. In our analysis, the training datasets consistently attained high scores across all metrics. The test datasets also achieved solid scores, though slightly lower and without significant loss, thus indicating that overtraining was not present. As seen in Figure 3 and Figure 4, the proposed XGB model achieved a larger area in both the training and test phases, thus demonstrating its balanced performance. The algorithms closest to our proposed model were RF and AdaBoost. Regarding the GNB system, it showed lower performance in prediction.

**Figure 5:**
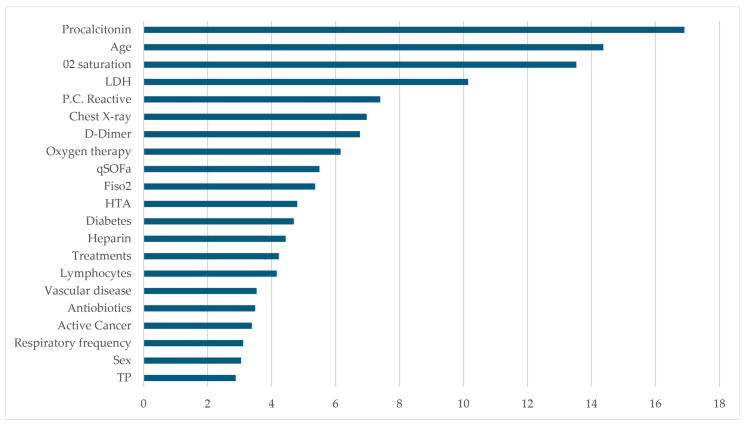
The figure depicts a histogram illustrating the most relevant parameters contributing to the prediction of mortality among emergency COVID-19 patients.

The proposed XGB system assigned weights to each variable for predicting mortality in COVID-19 cases. Among these variables, elevated procalcitonin, age, and initial oxygen saturation in the emergency department received the highest weights, as shown in Figure 5. Immediately following were variables such as lactate dehydrogenase (LDH), C-reactive protein, the presence of infiltrates in chest radiography, and D-dimer. Additionally, our system identified the importance of the patient’s need for oxygen therapy and a high score on the quick Sepsis Related Organ Failure Assessment (qSOFA) scale, which is widely used for early sepsis diagnosis. Other comorbidities such as hypertension and diabetes also contributed moderately to our prediction.

In this study, the Big-O notation technique has been used to analyze the complexity of the implemented algorithms. This technique allows for the calculation of the complexity and performance of each system [72]. As shown in Table 5, the proposed system exhibited logarithmic growth O(log(N)). Other systems that achieved similar growth include AdaBoost and Random Forest (RF). Conversely, the algorithm with the worst performance in terms of complexity was the SVM. The KNN and CNN models reached complexity values of O(N).

## 5. Discussion

The implementation of artificial intelligence in emergency services can provide significant assistance for rapid and accurate diagnosis, resource optimization, and improved effectiveness in clinical decision making within a short period of time. These AI systems have the ability to learn and improve over time as they process more data and receive feedback, thus making them valuable in emergency services to quickly assess all patient characteristics and provide an initial approximation of the medical treatment they might receive, thereby optimizing clinical outcomes. This application can be crucial in pandemic management [1,2,73].

Since the onset of a pandemic like COVID-19, understanding the behavior of the microorganism, in this case SARS-CoV-2, becomes the top priority in attempting to predict the mortality and morbidity of the disease. Due to this unfamiliarity, numerous studies initially emerged comparing clinical characteristics, risk factors, and outcomes among hospitalized COVID-19 patients with other respiratory viruses that are better understood and managed by current medicine. The aim was to anticipate the progression of the new emerging pathogen. Early comparisons were often made with the influenza virus due to their similarities [74,75,76].

Additionally, overlapping with periods of outbreaks of other respiratory viruses, such as the respiratory syncytial virus (RSV), COVID-19’s potential influence on the epidemiology of these viruses has also been studied [77]. It could even be possible that prior infection with respiratory viruses such as rhinovirus enhances the immune response to subsequent exposure to SARS-CoV-2, thus aiding in more effective viral clearance, as proposed by Radzikowska et al. [78]. Conversely, measures like quarantine implemented during the COVID-19 era may have influenced the behavior of other respiratory viruses afterward, as suggested by Olsen [79] below. In any case, the emergence of an infectious disease behaving like a pandemic has such extensive effects that leveraging technology to anticipate all preventive measures can be a significant advantage, with hospital emergency services being the first line of defense.

Once the behavior of the microorganism is understood, efforts are made to predict its evolution. Traditionally, clinical data and laboratory values have been analyzed primarily to predict the need for hospitalization as a severity criterion, the requirement for ICU admission, and/or the need for invasive mechanical ventilation therapy using conventional statistics, with an accuracy ranging from 86% to 88% [16].

Just as other similar microorganisms are used as models when an infectious disease behaves like a pandemic, if we take COVID-19 as an example, implementing these tools to rapidly create predictive models that help optimize resources could be applied in the future with other potentially serious pathogens for the same purpose. Moreover, if we can utilize artificial intelligence, for instance, to expedite triage tasks, healthcare professionals who previously performed this work could focus on providing medical care to the most critical patients as quickly as possible, thus leading to improved vital prognosis. Learning from the past to optimize the future is crucial, and this study underscores its importance.

Based on the literature review, studies began to emerge for predicting mortality in COVID-19 patients. For instance, Yadaw et al. [80] tested the utility of four ML algorithms using a wide dataset (n = 3841) to predict COVID-19 mortality. While these authors achieved high accuracy (area under the curve of 0.91), our model achieved 0.94. Another example is the study by Gao Y et al. [81], which presented a model to stratify the risk of death from COVID-19 based solely on clinical characteristics using four ML algorithms, including logistic regression, SVM, DT, and Neural Network (NN), thus achieving a similar area under the curve value. Similarly, An C et al. [82] trained four ML techniques on data from 10,237 patients, with their SVM achieving a specificity of 91.4%, a sensitivity of 90.7%, and an ROC of 0.96, thereby also obtaining a comparable area under the curve value. Other authors like Moulaei et al. also predicted COVID-19 mortality, thus concluding that Random Forest was the best model, wherein they achieved an area under the curve of 1 but with data from 850 patients [83]. Our proposed XGB model obtained higher accuracy with a smaller number of patients. The differences in results between a CNN and XGB on structured data stem from the inherent characteristics and capabilities of each model. CNNs are optimized to capture complex patterns in data such as images or sequences by using deep learning to extract hierarchical features. However, in structured tabular data, where relationships between variables are more direct and feature interpretation is crucial, XGB and other tree-based models like RF are typically preferred. These models are more effective at handling high dimensionality, data sparsity, and providing a clearer interpretation of how features influence predictions, thereby adapting better to problems where interpretability and the ability to handle nonlinear relationships are essential.

The behavior of SARS-CoV-2 was indeed peculiar, especially in the initial stages when clinicians faced patients exhibiting significant disparities between clinical severity and the objective analytical and imaging data obtained. These peculiarities persisted for an extended period until epidemic control was achieved. We do not know how future pandemics caused by other pathogens will unfold until they arise, as seen with the influenza A virus, dengue, or any other emerging pathogen. We believe our predictive model could prove beneficial for managing any of these or future pandemics, thus operating from emergency services.

Another notable benefit of machine learning methodology is its capability to leverage numerous variables with diverse characteristics. This capability is not a hindrance but rather an advantage in developing predictive models, thus enriching them, enhancing result accuracy, and broadening their applicability. In our study, we opted for the XGB method due to its exceptional scalability and rapid execution speed, which are key factors contributing to its success in ML applications. Furthermore, machine learning approaches allow multiple variables and their complex interactions to be tested simultaneously to create predictive models [84,85,86]. When applied to medicine, this makes machine learning a crucial tool for developing these systems for various diseases in the future. In biomedical fields, XGB has been previously used to classify patients with cancer [69,87], lupus [88], epilepsy [89], and chronic kidney [90] disease, and we believe it could be useful for any other type of emerging and nonemerging disease, although more studies and algorithm refinement are needed. The XGB model is capable of assigning weight to each variable according to its importance in predicting COVID-19 mortality, which sets it apart from other models. We obtained a significant weight for procalcitonin value, age, and oxygen saturation.

Once a patient arrives at the emergency department, unlike other models, the XGB system can assign a weight to each variable based on its importance in predicting COVID-19 mortality. Through data analysis, we found significant weights for procalcitonin levels, age, and oxygen saturation. Immediately following were variables such as LDH, C-reactive protein, the presence of infiltrates on chest X-ray, and D-dimer. This is crucial, because knowing these variables quickly can enhance decision making regarding which patients have poorer prognoses and should be admitted or which patients can be discharged. Similarly, understanding the weight of these variables can guide studies to determine effective treatments to halt disease progression, such as investigating the efficacy of anticoagulation therapy when D-dimer elevation first suggested worse prognosis, as studied by Moreno [91], or the emergence of thromboembolic disease as a complication of COVID-19 in emergency departments, as examined by Rodriguez [92]. This underscores the importance of identifying these variables as early as possible.

In addition, our system also identified the importance of the patient’s need for oxygen therapy. This could be crucial for hospital resource preparation, such as bed allocation or even increasing their number considering the demand for oxygen supply. Such preparation could prevent shortages in healthcare provision due to material scarcity, thereby eliminating the need for patient transfers to other healthcare facilities to mitigate these deficiencies. Combined with the predictive capability of the XGB system, this would enable us, based on a pandemic like COVID-19, to derive new prognostic variables to guide initial steps in combating a new pandemic.

In studies throughout 2021, age and oxygen saturation were also shown to be key factors in the mortality of these patients, as analyzed by Losonczy G et al. [93]. We were surprised by the importance the system assigns to the procalcitonin variable in COVID-19 patient mortality, which could be interpreted due to the possible coexistence of bacterial infections in patients with viral infections, as described by Jennie Han et al. [93]. This logically increases the severity of the patient and worsens their prognosis, thus emphasizing the importance of early treatment, as procalcitonin is a well-known marker in emergency services [94].

A mortality prediction model was used with clinical, analytical, and radiological data [21,23]. It was found that the use of ML could achieve better performance more quickly for the prediction of such emergency patients. The first wave of COVID-19 and AI was used for analysis without the use of currently recognized drugs. This idea provides us with a demonstration for future previously unknown emerging diseases, where the speed of results is necessary compared to the traditional statistical system.

The recent COVID-19 pandemic has taught us that all healthcare systems need to be organized and prepared to respond to any infectious disease. In this regard, the threat of a new pandemic will always loom. During the World Government Summit, the Director General of the World Health Organization (WHO), Tedros Adhanom Ghebreyesus, predicted that there will be another pandemic, although it is uncertain when, and it could be caused by another respiratory virus (such as influenza, another coronavirus, or an unknown disease). He also acknowledged that the world “remains unprepared for a new pandemic” [95].

Since mid-2023, the WHO has issued a statement urging all countries to prepare for new emerging global threats. The measures proposed include increased investment, promoting coordination and cooperation among countries, and emphasizing priority actions based on past events. During health crises like pandemics, there is a risk of emergency services becoming overwhelmed, which is why triage systems exist to prioritize patients based on severity. Currently, the five most important triage models worldwide are the following: the Canadian Emergency Department Triage and Acuity Scale (CTAS), the Australasian Triage Scale (ATS), the Emergency Severity Index (ESI), the Manchester Triage System (MTS), and the Sistema Español de Triaje (SET). These models assess specific variables related to airway, breathing, circulation, neurological disability, and patient exposure to categorize emergency services into five priority levels. These variables are assessed and inputted by healthcare personnel.

Our goal is to use AI to develop a system that, as it gathers data, can self-train to quickly detect patients at potential risk of transmitting infectious diseases during emergencies. This system aims to initiate early organizational prevention measures such as placing patients in individual isolation rooms, ensuring mask use, proper hand hygiene, and even employing Personal Protective Equipment (PPE). This approach would enhance healthcare professionals’ safety and disrupt the transmission chain among patients. Therefore, implementing our AI model would introduce a novel system to enhance emergency triage capacity and ensure that healthcare professionals are equipped with appropriate protective measures to mitigate contagion risks. It could seamlessly integrate with existing triage models used worldwide in emergency services.

Other advantages of this triage method would include the quicker identification of critically ill patients, improved prioritization, optimized patient flow, the prevention of emergency department overcrowding, and, ultimately, the enhancement of medical care quality.

Compared to other machine learning algorithms, the proposed model based on XGBoost 2.1.0 (XGB) offers several significant advantages. XGB is renowned for its ability to efficiently handle complex and large datasets due to its optimized implementation and capability to parallelize the training process. Moreover, XGB can manage a variety of data types and variables, including numeric and categorical, without requiring extensive preprocessing. This versatility makes it suitable for a wide range of applications.

Another key advantage of XGB is its ability to capture nonlinear and complex relationships among variables, which is achieved through its sequential tree-building approach that incrementally improves the model. This boosting approach enhances model accuracy by focusing on residual errors from previous iterations, which is particularly beneficial in problems where the relationships between features and the target variable are challenging to model linearly.

Additionally, XGB provides built-in tools for feature importance selection and model evaluation, thus facilitating the interpretation and optimization of the final model. Its capability to handle imbalanced data and resistance to overfitting are also standout features that make it preferred in scenarios where generalization and accuracy are critical.

The proposed system was compared with different machine learning methods described in the literature, as shown in Table 3 and Table 4. The comparison of the systems demonstrated a significant improvement in XGB compared to the other methods studied. The GNB and BLDA methods yielded lower performance than the other systems across all analyzed parameters, with values close to AUC = 76 and recall = 80%. The method that came closest to the precision values of the proposed method was RF, which achieved an AUC value of 89% and recall = 89%. It is noteworthy that the proposed method achieved a balanced radar plot between the training and test phases. This results in a reliable tool that facilitates automatic analysis to assist in predicting mortality in future pandemics.

A limitation of our study could be the wide diversity among the collected patients. We acknowledge the variability, ranging from critically ill to minimally symptomatic individuals in our series, which could introduce bias. However, we consider this diversity potentially advantageous for future pandemics, as the status of patients—whether critical or noncritical—can change rapidly. Additionally, one can consider the preservation some physical structures and physical properties, such as long time behavior, maximum principle, singular solutions, and positivity preservation [96,97,98].

## 6. Conclusions

The aim of this study is to create a useful and innovative tool to streamline triage systems in hospital emergency services using machine learning algorithms and to identify the most predictive variables in the process.

Procalcitonin has proven to be a significant predictor of mortality in COVID-19 patients. Other clinical, analytical, and radiological factors include age, initial oxygen saturation in the emergency department, LDH levels, C-reactive protein, and chest X-ray with peripheral interstitial infiltrates—in that order.

These findings underscore that XGBoost (XGB) is a valuable and innovative tool in emergency triage systems to guide patient care pathways during future pandemics, thus drawing from the example of COVID-19. Therefore, applying artificial intelligence for COVID-19 and future pandemics can significantly enhance detection, response, and disease management capabilities, thereby saving lives and mitigating public health impacts.

## Figures and Tables

**Table 1:** Main hyperparameters of the machine learning algorithms evaluated in the study.

Method	Parameters
SVM	Kernel function: Gaussian
	Sigma = 0.5
	C = 1.0
	Numerical tolerance = 0.001
	Iteration limit = 100
DT	Minimum number of instances in leaves = 4
	Minimum number of instances in internal nodes = 6
	Maximum depth = 100
BLDA	Kernel: Bayesian
NN	Number of hidden layers: 2 layers.
	Max neurons per hidden layer: 64.
	Activation function: ReLU.
	Learning rate: 0.001.
	Batch size: 64.
	Number of epochs: 100.
	Regularization: L2 regularization (Ridge)
	Weight initialization: Glorot/Xavier initialization.
GNB	Usekernel: False
	fL = 0
	Adjust = 0
CNN	Learning rate = 0.1
	Network section depth = 3
	Pooling type: Max pooling.
	Momentum = 0.9
	Pool size: 64
	L2 regularization = 1 × 10^−3^
Adaboost	Base estimator: tree
	Maximum number of splits = 20
	Learning rate = 0.1
	Number of learners = 50
KNN	Number of neighbors = 20
	Distance metric: Euclidean
	Weight: Uniform
XGB	Eta = 0.20
	Minimum chil weight = 1
	Maximum depth = 7
	Number of learners = 50
	Maximum delta step = 3

**Table 2:** The table presents the epidemiographic data and emergency department (ED) onset symptomatology of the study.

	n	%
Male sex	225	37.19
HTA	318	52.56
Type 2 DM	153	25.29
EPOC	54	8.93
Severe asthma	16	2.64
ERC	40	6.61
Obesity	45	7.44
Pregnancy	1	0.16
Dyslipemia	149	24.63
Liver disease	9	1.49
ETV	9	1.49
Active cancer	27	4.46
Institutionalized	50	8.26
Cough	378	62.48
Fever	438	72.4
Dyspnea	327	54.05
Chest pain	20	3.31
Myalgia	98	16.2
Headache	10	1.65
Anosmia	19	3.14
Ageusia	26	4.3
Diarrhea	65	10.74
Asthenia	136	22.48
Admission	495	81.82
Exitus	132	21.82

**Table 3:** The table displays the average values and standard deviations of accuracy, recall, Kappa, and precision.

Methods	Accuracy (%)	Recall (%)	Kappa (%)	Precision (%)
SVM	83.74 ± 0.87	83.84 ± 0.85	73.77 ± 0.86	83.15 ± 0.85
BLDA	79.96 ± 0.92	80.06 ± 0.91	71.04 ± 0.90	79.36 ± 0.92
DT	82.65 ± 0.78	82.75 ± 0.79	72.93 ± 0.77	82.13 ± 0.78
GNB	75.59 ± 0.98	75.68 ± 0.97	67.36 ± 0.95	75.12 ± 0.96
NN	84.24 ± 0.73	84.01 ± 0.75	74.53 ± 0.74	84.58 ± 0.73
KNN	85.96 ± 0.68	86.09 ± 0.71	76.36 ± 0.69	85.70 ± 0.68
CNN	84.97 ± 0.71	85.04 ± 0.75	75.23 ± 0.73	85.02 ± 0.73
AdaBoost	88.53 ± 0.77	88.64 ± 0.74	78.82 ± 0.76	87.90 ± 0.75
RF	89.14 ± 0.65	89.25 ± 0.69	79.42 ± 0.67	88.51 ± 0.66
XGB	91.62 ± 0.47	91.71 ± 0.45	82.53 ± 0.46	90.97 ± 0.45

**Table 4:** The table presents the average values and standard deviations of AUC, F1 score, MCC, and DYI.

Methods	AUC	F1 Score (%)	MCC (%)	DYI (%)
SVM	0.84 ± 0.02	83.49 ± 0.84	74.31 ± 0.85	83.74 ± 0.85
BLDA	0.80 ± 0.02	79.71 ± 0.92	70.94 ± 0.91	79.96 ± 0.92
DT	0.83 ± 0.02	82.44 ± 0.79	73.39 ± 0.77	82.65 ± 0.78
GNB	0.76 ± 0.02	75.40 ± 0.98	66.51 ± 0.96	75.59 ± 0.97
NN	0.84 ± 0.02	84.46 ± 0.80	75.32 ± 0.78	84.45 ± 0.79
KNN	0.86 ± 0.02	85.90 ± 0.72	76.18 ± 0.75	85.96 ± 0.73
CNN	0.85 ± 0.02	85.17 ± 0.76	75.93 ± 0.73	85.01 ± 0.76
AdaBoost	0.88 ± 0.01	88.27 ± 0.72	78.56 ± 0.74	88.53 ± 0.73
RF	0.89 ± 0.01	88.91 ± 0.67	79.10 ± 0.68	89.13 ± 0.67
XGB	0.92 ± 0.01	91.34 ± 0.46	83.02 ± 0.45	91.62 ± 0.46

**Table 5:** The complexity of the classification algorithms as determined by the Big-O notation.

Method	Number of Samples N				Big-O
	10^4^	2 × 10^5^	5 × 10^6^	10^7^	
SVM	2634	5550	20,770	351,681	O(N²)
BLDA	3565	6980	13,970	27,470	O(N)
DT	3883	7169	9436	11,703	O(log(N))
GNB	3161	6459	12,759	26,349	O(N)
RF	3345	4468	5695	9097	O(log(N))
NN	3225	6898	12,224	25,361	O(N)
KNN	2307	4824	10,479	23,945	O(N)
CNN	5660	9689	16,407	28,736	O(N)
AdaBoost	3008	4358	7067	9312	O(log(N))
XGB	2080	3002	4358	4413	O(log(N))

## Data Availability

The datasets used and/or analyzed during the present study are available from the corresponding author upon reasonable request.
